# Cross-Talk between the TGF-β and Cell Adhesion Signaling Pathways in Cancer

**DOI:** 10.7150/ijms.96274

**Published:** 2024-05-13

**Authors:** Jiahao Liao, Rentang Chen, Bihua Lin, Runhua Deng, Yanfang Liang, Jincheng Zeng, Sha Ma, Xianxiu Qiu

**Affiliations:** 1 Guangdong Provincial Key Laboratory of Medical Molecular Diagnostics, Dongguan Key Laboratory of Medical Bioactive Molecular Developmental and Translational Research, The First Dongguan Affiliated Hospital, Guangdong Medical University, Dongguan, Guangdong, 523808, China.; 2 Institute of Laboratory Medicine, School of Medical Technology, Guangdong Medical University, Dongguan, Guangdong, 523808, China.; 3 Department of Pathology, Binhaiwan Central Hospital of Dongguan, Dongguan, Guangdong, 523905, China.; 4 School of Biomedical Engineering, Guangdong Medical University, Dongguan, Guangdong, 523808, China.

**Keywords:** TGF-β signaling pathway, cell adhesion signaling pathway, cancer

## Abstract

Transforming growth factor-β (TGF-β) is strongly associated with the cell adhesion signaling pathway in cell differentiation, migration, etc. Mechanistically, TGF-β is secreted in an inactive form and localizes to the extracellular matrix (ECM) via the latent TGF-β binding protein (LTBP). However, it is the release of mature TGF-β that is essential for the activation of the TGF-β signaling pathway. This progress requires specific integrins (one of the main groups of cell adhesion molecules (CAMs)) to recognize and activate the dormant TGF-β. In addition, TGF-β regulates cell adhesion ability through modulating CAMs expression. The aberrant activation of the TGF-β signaling pathway, caused by abnormal expression of key regulatory molecules (such as Smad proteins, certain transcription factors, and non-coding RNAs), promotes tumor invasive and metastasis ability via epithelial-mesenchymal transition (EMT) during the late stages of tumorigenesis. In this paper, we summarize the crosstalk between TGF-β and cell adhesion signaling pathway in cancer and its underlying molecular mechanisms.

## Introduction

Transforming growth factor-β (TGF-β) was initially isolated as a tumor factor from mouse sarcoma cells and was found to induce certain non-tumor cells to form growth colonies on soft agar 1, 2. With the in-depth study of it, researchers gradually find that TGF-β has a dual role: as a tumor suppressor, TGF-β inhibits cell proliferation and promotes cell apoptosis and aging to maintain the homeostasis of tissues and organs; on the other hand, as a tumor promoter, TGF-β promotes tumor cell proliferation, transformation, invasion, and metastasis of tumor cells, which plays a key role in tumorigenesis and development 3. As the common characteristic of malignant tumors, invasion and metastasis often involve changes in cell adhesion mechanisms 4, which include EMT, remodeling of the ECM, and loss of cell-cell adhesion 5.

It is reported that TGF-β is highly relevant to the cell adhesion mechanisms 6. TGF-β is stored in an inactive form on the ECM and can be activated by CAMs through specific binding. The release of TGF-β from ECM sites greatly enhances the TGF-β signaling pathway 7. Additionally, TGF-β can regulate the synthesis, deposition, and remodeling of ECM components 8 and the expression of certain CAMs in cells and the extracellular matrix 9.

Cell adhesion is a fundamental mechanism shared by all multicellular organisms, allowing individual cells to assemble into a three-dimensional structure. A variety of cell adhesion mechanisms determine the diversity and specificity of tissues, which are characterized by cell-cell and cell-extracellular matrix connections. Normally, cell adhesion systems are in a dynamic process, not only maintaining the homeostasis of the tissue structure, but also forming a complex signaling network that regulates various aspects of cellular activities, including proliferation, differentiation, motion, etc. 11. The cross-regulation of three systems, namely, the extracellular matrix, cell adhesion molecules, and cell-secreted cytokines or growth factors, plays an essential role in signaling networks 12.

Current studies have highlighted the interplay between the TGF-β and cell adhesion signaling pathway in tumors. The functional integrity and crosstalk between these two signaling systems are essential for maintaining tissue and organ homeostasis. In this paper, we review the extensive crosstalk between these pathways and the potential underlying molecule mechanism and discussed the role of the TGF-β signaling pathway in tumor development.

## The TGF-β signaling pathway

### The synthesis, secretion, and localization of TGF-β

TGF-β is a member of the TGF-β superfamily, and there are three TGF-β isoforms in mammals: TGF-β1, TGF-β2, and TGF-β3. These isoforms exhibit a high level of protein sequence conservation. Interestingly, knockout mice of each isoform display distinct phenotypes, indicating the functional diversity of these three isoforms 13-15.

As a multifunctional cytokine that is ubiquitously expressed in almost every tissue and cell type, TGF-β is involved in various processes including immune regulation, wound healing, and the development of tissues and organs. It is also crucial in maintaining tissue homeostasis and plays a role in the pathogenesis of diseases like cancer, by regulating cell growth, proliferation, differentiation, and apoptosis.

**Figure 1:**
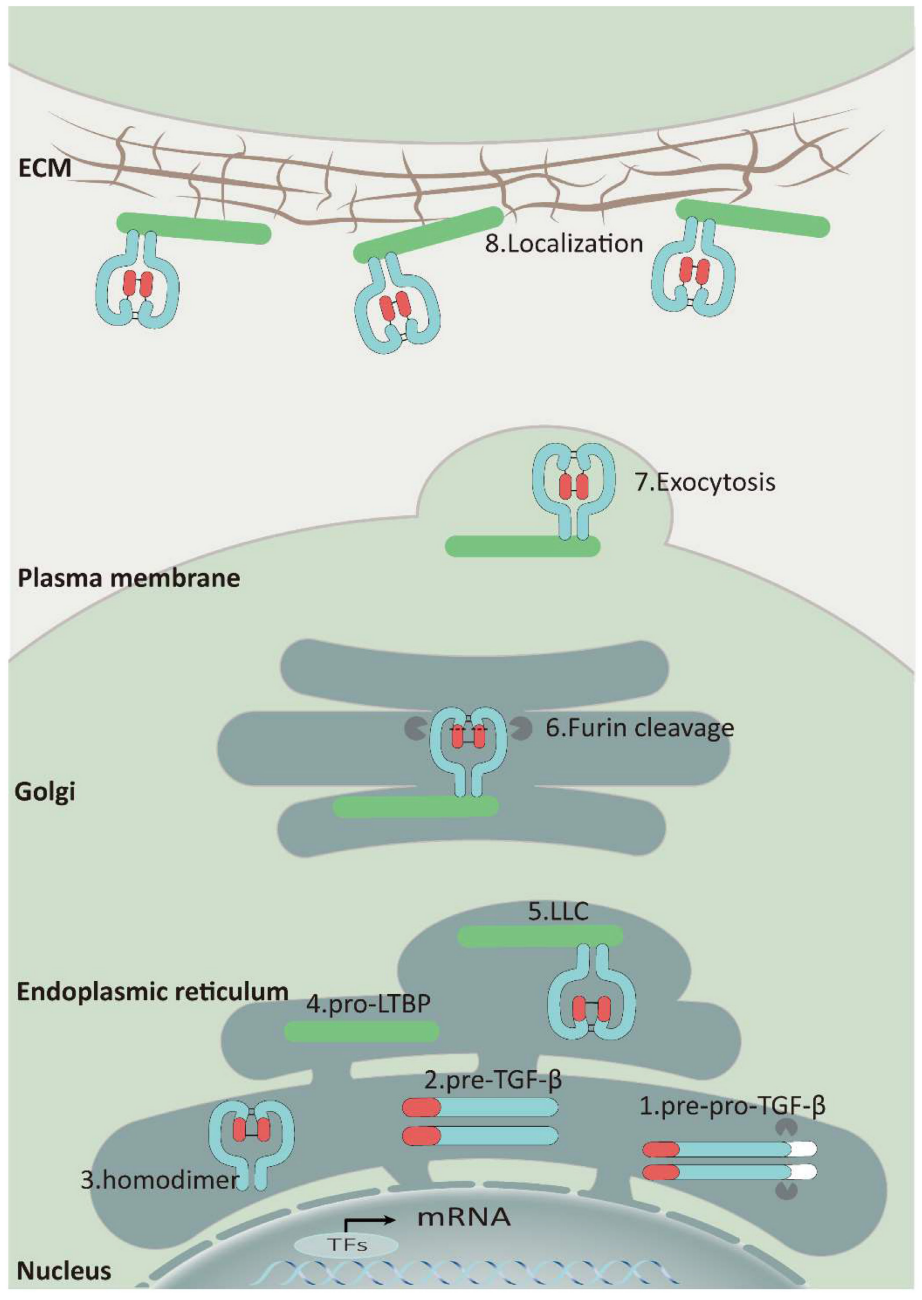
The Synthesis, Secretion, and Localization of TGF-β: Upon entry into the endoplasmic reticulum, the signal peptide of pre-pro-TGF-β (1) is rapidly cleaved to generate pro-TGF-β monomers (2), and then these monomers fold and form a homodimer (3) through disulfide bonds. Subsequently, the homodimer binds to LTBP (4) to form a ternary complex (5) and is translocated to the Golgi. Although TGF-β is released by furin, latency-associated protein (LAP) can still connect with TGF-β via non-covalent binding and form LLC that would be secreted (6) (7). Finally, LLC binds to fibronectin, fibronectin, *etc*. via LTBP and attaches to the ECM (8).

As a secreted protein, TGF-β is synthesized as a pre-pro-TGF-β form in ribosomes. This precursor consists of a small N-terminal signal peptide, a prodomain known as the latency-associated polypeptide, and a C-terminal mature polypeptide. Guided by the signal peptide, the nascent protein is transferred to the endoplasmic reticulum and cleaved, generating pro-TGF-β monomers. These monomers then fold and form a homodimer through disulfide bonds, creating a pro-TGF-β structure. This complex is transported to the cis-Golgi apparatus, where furin or furin-like proteinases cleave the prodomain, releasing the mature TGF-β cytokine 16. Nevertheless, latency-associated protein (LAP) can still form a complex with TGF-β through non-covalent binding, preventing it from binding to the TGF-β receptors known as the small latent complex (SLC) (Figure 1) 17.

Furthermore, SLC can form a large latent complex (LLC) by covalently attaching to the latent TGF-β binding protein 1,3,4 (LTBP1,3,4) through a pair of disulfide bonds in the endoplasmic reticulum. Most cells release LLC outside of the cell as an inactive form, which is attached to ECM by LTBPs binding to fibrillin, fibulins, fibronectin, and other ECM proteins (Figure 1) 18-20. The interaction between LAP and LTBPs facilitates the correct folding of TGF-β precursor proteins and is crucial for TGF-β activation 21. When a specific cysteine residue (C33) in the binding site of LAP to LTBPs is substituted with serine *in vivo*, mice exhibit inflammatory response and tumor characteristics that resemble those observed in TGF-β1-deficient mice 22.

### The TGF-β signaling pathway

**Figure 2:**
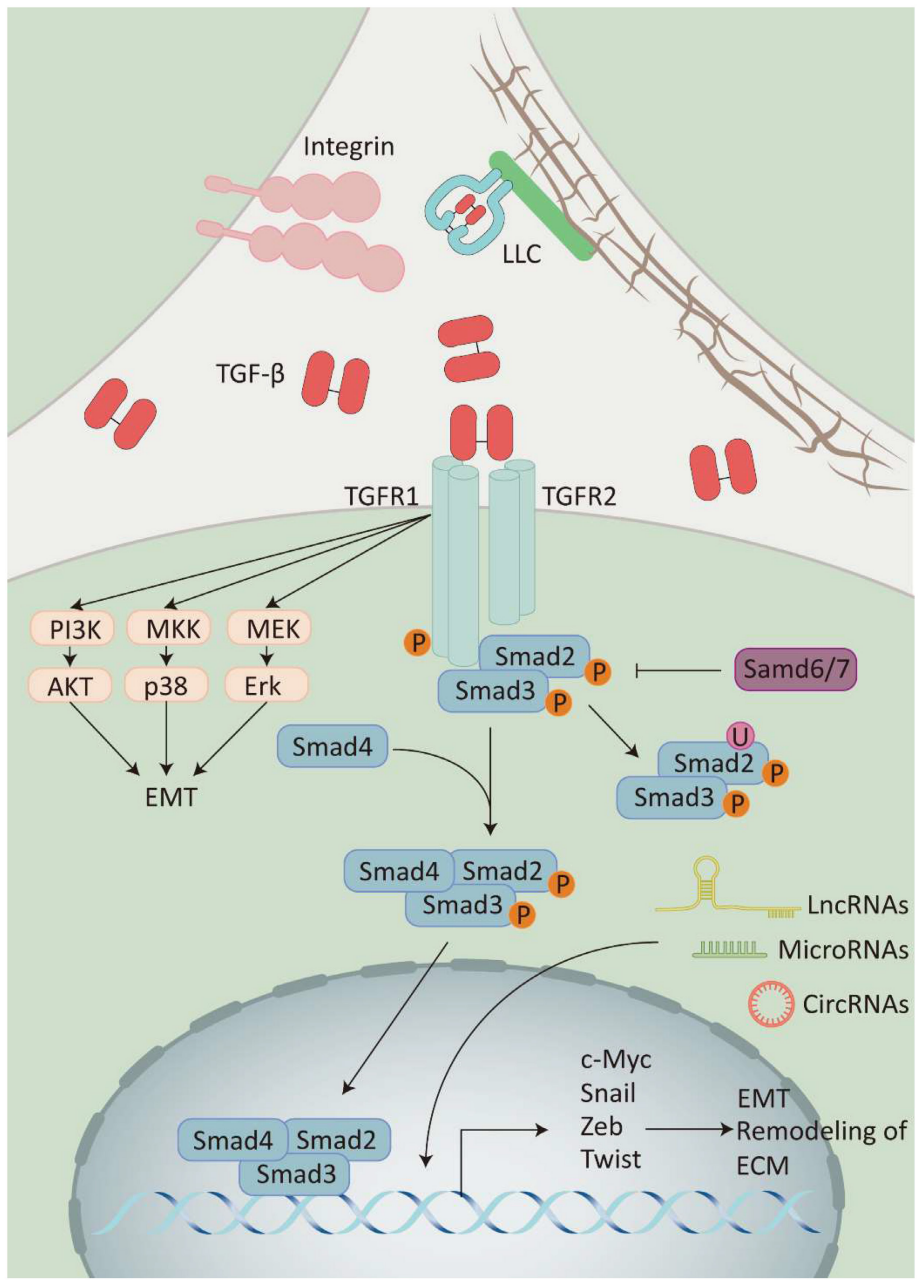
The TGF-β signaling pathway: Integrins release TGF-β by binding to LLC on ECM. TGF-β activates receptor-regulated Smad (Smad2/3) by recruiting the receptor(TβRI/II) and then binds to co-mediator Smad (Smad4) to activate the expression of target genes, while inhibitory Smad (Smad6/7) inhibits this process. TGF-β can also activate different signaling pathways to participate in the signaling pathway modification, including the PI3K/AKT pathway, the p38 MAPK signaling pathway, and the Erk1/2 pathway. Additionally, non-coding RNAs (LncRNAs, MicroRNAs, and CircRNAs) can also participate in the TGF-β signaling pathway by regulating the expression of TGF-β transcription factors.

Classical TGF-β signaling enables cell membrane-to-nucleus message transduction through the Smad protein family 23. At first, the activated TGF-β binds to the TGF-β type II receptor homodimer (TβRII) located on the cell membrane. This interaction leads to the recruitment and activation of the TGF-β type I receptor homodimer (TβRI) and then forms a heterotetramer. Subsequently, the activated TβRI phosphorylates the receptor-regulated Smad (R-Smad), which facilitates binding the R-Smad complex to co-mediator Smad (Co-Smad) to form a trimeric complex. Finally, the trimeric complex is transported and accumulates in the nucleus, acting as a transcription factor which enhances or inhibits the production of TGF-β target genes (Figure 2) 24.

Furthermore, activated TGF-β transmits signals through various signaling pathways, including phosphatidylinositol-3 kinase (PI3K)/AKT pathway, Rho/Rho-kinase pathway, and mitogen-activated protein kinase (MAPK) pathway, which includes extracellular signal-regulated kinase 1/2 (Erk1/2) pathway, p38 mitogen-activated protein kinase (p38 MAPK) pathway, and c-Jun amino-terminal kinase (JNK). These pathways are collectively known as non-classical TGF-β signaling pathway 25, 26. It has been shown that the diversity of TGF-β signaling depends on the combinatorial utilization of core pathway components such as ligands, receptors, Smads, and transcription factors. This diversity is further modulated by crosstalk with other signaling pathways and the integration of multiple signaling modules beyond TGF-β receptor-activated Smads, collectively regulating the transcription of target genes.

The PI3K/AKT pathway is initiated by the activation of PI3K, which phosphorylates phosphatidylinositol 4,5-bisphosphate (PIP2) to generate phosphatidylinositol 3,4,5-trisphosphate (PIP3). PIP3 serves as a second messenger that recruits and activates various downstream effectors, including the serine/threonine kinase AKT (also known as protein kinase B, PKB) 27. The crosstalk between the TGF-β signaling pathway and the PI3K/AKT signaling pathway frequently promotes EMT in epithelial malignant cancer. For instance, AKT-mediated Twist1 phosphorylation promotes breast cancer EMT and metastasis by modulating its transcriptional target TGF-β2, leading to enhanced TGF-β receptors signaling, which in turn maintains hyperactive PI3K/AKT signaling 28. In BxPC-3 pancreatic cancer cells, TGF-β1 promotes TGF-β1-induced EMT by enhancing the phosphorylation of AKT 29. The MAPK pathway is activated by a range of input signals, such as cytokines, chemokines, growth factors, and stress signals. For instance, TGF-β can stimulate the p38 MAPK signaling pathway, thereby augmenting the migratory and invasive capacities of cancer cells in non-small cell lung cancer 30. Within the liver cancer cells' tumor microenvironment, the cancer-associated fibroblasts (CAFs)-derived cardiotrophin-like cytokine factor 1 (CLCF1) increases TGF-β secretion in tumor cells. This, in turn, activates Erk 1/2 signaling in CAFs, leading to the production of additional CLCF1, creating a positive feedback loop that accelerates the development of liver cancer cells 31.

## The crosstalk between CAMs and TGF-β signaling pathway

The cell adhesion mechanism is regulated by the adhesion functional unit, CAMs, which play a crucial role in the interaction of cells to ECM and other cells 32. Characterized by their protein structures, CAMs can be divided into four groups: integrins, selectins, cadherins, and members of the immunoglobulin superfamily (IgSF) 33, 34. Among them, integrins are mainly related to cell-ECM interaction, while selectins, cadherins, and IgSF members are mainly involved in cell-cell adhesion 35.

Cell adhesion involves the interaction of cells with each other and with the ECM. It is fundamental to the organization of cells into tissues and organs, cell migration, and cellular signal transduction. CAMs function as transmembrane proteins, the extracellular domain stabilizes cells to bind to adjacent cells and ECM, while the intracellular domain interacts with the actin and/or cytoskeleton, allowing signals to be transmitted from outside to cells (outside-in signaling) 36. The outside-in signaling regulates cell adhesion ability and responds to the microenvironment 37. Additionally, CAMs can interact with transformed growth factors, transcription factors, and other signal proteins that participate in more extensive signaling pathways to mediate cell fate 38, 39. In the following discussion, we primarily focus on how integrins, cadherins, and IgSF are involved and regulate the TGF-β signaling pathway (Table 1).

### The crosstalk between TGF-β and integrins

Although latent TGF-β is widespread in many tissues and most cells have TGF-β receptors, TGF-β signaling requires mature TGF-β to bind to the receptors. Thus, a key element in controlling the TGF-β signaling pathway is knowing how to release mature TGF-β from the latent form of TGF-β 40. *In vitro*, it is relatively easy to induce conformational changes of latent TGF-β that lead to the release of mature TGF-β (i.e., TGF-β activation). For instance, purified latent TGF-β can be activated by acidic or alkaline pH, thermal denaturation, ionizing radiation, and oxidation of reactive oxygen species (ROS) 41-43. In contrast, the role of these activation pathways *in vivo* has not yet been elucidated. Current research shows that the essential activation mechanisms of TGF-β *in vivo* are primarily triggered by interaction with integrins (Figure 2) 43.

The integrins family are transmembrane heterodimer receptors on the cell surface and consist of 18 α-subunits and 8 β-subunits that can combine to generate 24 distinct integrins, 18 of which have ECM receptors activities. Integrins can be categorized into three primary groups based on ligand specificity: recognize the Arg-Gly-Asp (RGD) peptide receptors, collagen receptors, and laminin receptors 44, 45. Among these, integrins that bind to RGD have been extensively researched. The αvβ1, αvβ3, αvβ5, αvβ6, and αvβ8 receptors could attach to the LAP. Nevertheless, the activation of TGF-β is mainly mediated by integrins αvβ6 and αvβ8 16.

#### The crosstalk between TGF-β and integrin αvβ6

αvβ6 is an integrin that is mainly found in epithelial cells and upregulated following epithelial damage. Integrin αvβ6, unlike other RGD integrins, can recognize not only the RGD sequence, but also a specific LXXL/I motif. This motif forms an amphipathic α-helix that fits into a hydrophobic pocket made up entirely of residues from the β6 subunit. Thus, integrin αvβ6 exhibits a significantly strong affinity to pro-TGF-β 46. Integrin αvβ6 selectively triggers the activation of TGF-β1 and TGF-β3 while excluding TGF-β2. The integrin αvβ6 is absent in mice lacking the gene responsible for generating the β6 integrin subunit (Itgβ6 ^-/-^) because β6 exclusively binds to αv 47. There was no significant difference in the overall quantity of TGF-β protein in the lung tissue of Itgβ6 ^-/-^ and Itgβ6 ^+/+^ mice. However, in the bleomycin-induced lung fibrosis model, which is known to be heavily dependent on TGF-β activity, Itgβ6 ^-/-^ mice did not experience any significant effects following bleomycin treatment. Another study shows that the activation of TGF-β was observed by cell lines co-culture that express integrin αvβ6 and TGF-β receptors 48. Furthermore, the activation mechanism requires the association of integrin αvβ6 with the actin cytoskeleton through the cytoplasmic tail of the β6 subunit. In a mouse model of tendinopathy created based on excessive tensile strain, the presence of integrin αvβ6 triggers the activation of TGF-β1 in response to mechanical force. This activation leads to the degeneration of tendons, the formation of cartilage, and the growth of blood vessels, all of which contribute to the development of tendinopathy. External stretch or increased intracellular stress *in vitro* also directly activates TGF-β 49. These results indicate that the interaction between latent TGF-β and integrin αvβ6 is insufficient for activation. Activation of latent TGF-β requires the additional binding of integrin αvβ6 to the actin cytoskeleton.

The increased expression of integrin αvβ6 is strongly linked to tumor invasion, metastasis, and a poor prognosis in several human epithelial-derived malignancies 50, 51. In triple-negative breast cancer (TNBC), tumor cells could activate the TGF-β signaling pathway by αvβ6 integrin. This activation results in the increase of SOX4, which is an important regulator of immune evasion pathways 52. Colorectal cancer (CRC) cells that express integrin αvβ6 activate cancer-associated fibroblasts (CAFs) by TGF-β activation. CAF cells promote the migration of colorectal cancer cells to distant locations by activating the SDF-1/C-X-C chemokine receptor type 4 (CXCR4) axis 53. The co-expression of integrin αvβ6 with eukaryotic translation initiation factor 4E (eIF4E) or Ets-1 is regarded as a reliable prognostic marker of CRC 54, 55. Conversely, the concurrent presence of integrin αvβ6 and Matrix Metallopeptidase 9 (MMP-9) is a reliable predictive marker in individuals diagnosed with gastric cancer 56.

#### The crosstalk between TGF-β and integrin αvβ8

While integrin αvβ6 has been extensively studied, the adhesion and signaling roles of integrin αvβ8 in development, physiology, and disease are still yet to be investigated. It is suggested that integrin αvβ6 and integrin αvβ8 trigger TGF-β through distinct pathways. The activation of TGF-β by integrin αvβ8 occurs in a unique structural domain called β8. This domain lacks a cytoplasmic tail and only has a differentiated cytoplasmic structural domain 57. This domain does not interact with the actin cytoskeleton and exists in an extended closed structure 58, 59. The activation of TGF-β by integrin αvβ8 does not depend on actin cytoskeletal interactions and is continuous activation 60. In addition, integrin αvβ8 could interact with LAP in a local binding way and form a protein complex. This process does not rely on the release and spread of mature TGF-β but rather directly activates the TGF-β signaling pathway in the local area 61, differentiating it from other activation modes which involve the release and dispersion of mature TGF-β 62, 63.

The current studies indicate that developmental cerebral hemorrhage and postnatal microglia maturation arrest and activation occur in both Itgβ8 mutant and TGFβR1^-/-^ mice. These findings align with the characteristics observed in microglial cell lineage-specific conditional deletion of TGFβR2, implying that the central nervous system (CNS) microenvironment relies on the same molecular mechanism, namely the αvβ8-TGF-β signaling, to regulate the development and maturation of vascular and microglial cells 64, 65.

#### Integrins Participation in the TGF-β Signaling Pathway via Integrin Adhesome

Kindlin-1 is a protein that binds to the tail of integrin and enhances the activation of TGF-β through αvβ6 integrin. Kindler syndrome is caused by mutations or deletions of Kindlin-1 in human and mouse keratinocytes. The disease is characterized by skin blistering, premature skin aging, and an elevated risk of malignancy 66. Mouse keratinocytes lacking the Kindlin-1 gene experience significant disruptions in β6 integrins-dependent cell adhesion, spreading, assembly of F-actin stress fibers, and release of TGF-β. This leads to an increased risk of tumor development 67. Kindlin-2, a member of the kindlin family, also plays a role in the activation of TGF-β by integrins. In breast cancer, Kindlin-2 stabilizes the β1 integrin-TβRI complexes and facilitates the TGF-β signaling pathway 68. Kindlin-2 is also established as a requirement for BC tumor development and progression in transgenic mice 69.

### The crosstalk between TGF-β and Cadherins

Cadherins feature cadherin repeat sequences in their extracellular domain which are stabilized by calcium ions. Cadherins can be categorized into classical cadherins, protocadherins, desmosomal cadherins, and atypical cadherins 70. Several of them have a strong link with the TGF-β signaling pathway. Classic Cadherins, such as E-Cadherin and N-Cadherin, have a significant function in TGF-β-mediated EMT, as will be discussed later. Cadherin-11 is also a classical adhesion molecule expressed by mesenchymal stem cells (MSCs) and is necessary to induce the differentiation of MSCs. Cadherin-11 can regulate the expression of TGF-β1 and induce the differentiation of MSCs into contractile smooth muscle cells (SMCs) through TGF-β receptor II pathway. At the same time, Cadherin-11 can influence MSCs differentiation indirectly by regulating the ECM via the TGF-β1 pathway 71. Furthermore, in glioma tumors, FAT atypical cadherin 1 (FAT1) promotes the translation of TGF-β1/2 by inhibiting the level of miR-663a, forming an immunosuppressive microenvironment, which in turn facilitates the immune evasion of GBM 72.

### The crosstalk between TGF-β and IgSF

The IgSF is one of the largest and most diverse protein families 73. All members of the IgSF typically contain one or more immunoglobulin or immunoglobulin-like domains. Many IgSF proteins function as cell adhesion molecules that are regulated by TGF-β. For example, TGF-β induces lung injury by the overexpression of intercellular adhesion molecules (ICAMs) on endothelial cells, leading to neutrophil-mediated damage 74. TGF-β can also induce the expression and shedding of activated leukocyte cell adhesion molecule (ALCAM) and thereby promote bone metastasis of prostate cancer 75. In colorectal cancer, the high expression of TGF-β may inhibit the expression of vascular cell adhesion molecule 1 (VCAM-1) in tumor vasculature, which allows tumor cells to avoid immunosurveillance by circulating lymphocytes 76. TGF-β can induce the lysosomal pathway degradation of Junctional adhesion molecule A (Jam-A), a tight junction component facilitating epithelial cell-cell adhesion. This allows breast cancer cells to acquire invasive cell properties during the EMT process 77, 78.

## The crosstalk between ECM and TGF-β signaling pathway

The ECM is a complex network that provides structural and biochemical support to cells within tissues and organs 79. It consists of various structural proteins, polysaccharides, adhesion proteins, and other molecules secreted by cells and organized into a three-dimensional network surrounding cells 80, 81. TGF-β is mainly secreted and stored in the ECM as a latent complex 82. TGF-β activation in normal cells causes the transformation of myofibroblasts into fibroblasts, which promotes pro-healing homeostasis 83. TGF-β can regulate the remodeling of ECM components that facilitate tumor migration and invasion in the majority of tumor tissues. This remodeling is frequently accompanied by alterations in the expression of base proteinases (MMPs) 84, 85. By inhibiting Grhl2 expression, TGF-β can promote MMP-2, MMP-7, and MMP-9 expression in gastric cancer, thereby facilitating the invasion and migration of gastric malignant cells 86. TGF-β upregulates MMP-2 and MMP-9 expression in p38 MAPK signaling pathway that leads to the invasive and migratory phenotypes in MCF10A human breast epithelial cells 87. Specifically, TGF-β promotes the expression of MMP-2 by inhibiting the transcription factor ATF2 88. Additionally, it has been demonstrated that MT1-MMP can cleave LAP and is essential for αvβ8-mediated TGF-β activation 89. In human prostate and breast cancer cells, MT1-MMP promotes cancer cell EMT and migration by activating TGF-β 90.

## Regulation of TGF-β and EMT

The epithelial-mesenchymal transition is a crucial process that has a significant impact on both physiological and pathological events, including embryogenesis, tissue damage repair, and cancer progression 91. It is well known acknowledged that EMT is closely related to cell adhesion mechanism: alterations in various cell adhesion molecules, such as claudins and E-cadherin 92, induce the detachment of epithelial cells from each other and the underlying basement membrane 93. During this process, epithelial cells gradually acquire mesenchymal phenotypes (such as reduced intercellular adhesion and increased motility) in response to the microenvironment 94.

The initiation and development of EMT are affected by several signaling pathways, among which the TGF-β signaling pathway plays an important role 95. Under physiological conditions, TGF-β is involved in mesenchymal phenotypic transitions in epithelial and endothelial progenitor cells during development 23, and in wound healing, it promotes macrophage-mediated inflammation and tissue debridement 96. However, when the TGF-β signaling pathway is disturbed, it can promote tissue fibrosis and facilitate cancer cells' migration, invasion, and metastasis through EMT 3, 97, 98.

### Smads signaling in TGF-β-mediated EMT

TGF-β induces EMT by promoting the expression of transcriptional repressors of E-cadherin, such as Snail, Zeb, and Twist 99. As a prominent effector protein of the TGF-β signaling pathway, Smad is a central mediator of EMT in many contexts. In keratinocyte-specific Smad2-deficient mice, a decrease in E-cadherin and the activation of Snail were observed in the epidermis. Furthermore, Smad2 deficiency resulted in a notable increase in Smad4 binding to the Snail promoter. This led to an acceleration of chemically induced skin tumor formation and malignant progression 100. In primary renal tubular epithelial cells from Smad3-deficient mice, exogenous TGF-β1 treatment was unable to alter epithelial cell phenotypic characteristics, which resulted in Smad3-deficient mice being protected from tubulointerstitial fibrosis after unilateral ureteral obstruction (UUO) 101. Consistent with lung carcinoma cells 102, Smad3 deletion similarly inhibited the process of EMT. However, in gastric cancer cells, Smad3-mediated TGF-β signaling induces carcinoembryonic antigen (CEA)-related cell adhesion molecule 6 (CEACAM6) expression and promotes EMT in gastric cancer cells 103. In bronchial epithelial cells chronically exposed to toluene diisocyanate (TDI), TGF-β1 secretion was increased, and activation of the TGF-β signaling pathway through massive activation of Smad2/3. This led to a decrease in cell adhesion molecules and facilitated the process of EMT and the cancerous transformation of bronchial epithelial cells 104. Knockdown of Smad4 effectively inhibits TGF-β-induced EMT in normal mammary cells and strongly suppresses bone metastasis of breast cancer cells in nude mice 105. The Smad7-mediated negative feedback loop of TGF-β signaling was disturbed in some breast and lung cancer cell lines, while TGF-β-induced EMT and cancer cell invasion were reversed when Smad7 transcription was upregulated 106.

### c-Myc in TGF-β-mediated EMT

Myc is a group of proto-oncogenes that encode transcription factors, specifically c-Myc (MYC), I-Myc (MYCL), and n-Myc (MYCN) 107. Dysregulation of c-Myc activity is a common occurrence in many malignancies, resulting in the development of tumors and the perpetuation of the disease 108, 109. C-Myc is a crucial downstream target gene of the TGF-β signaling pathway and is widely recognized for its significant involvement in TGF-β-mediated EMT 110. For example, in pancreatic cancer cells, TGF-β regulates the expression of RAP2 via the transcription factor c-Myc. Promotes N-cadherin, and vimentin, and decreases E-cadherin in pancreatic cancer 111. Furthermore, quercetin also inhibits EMT by blocking the TGF-β signaling pathway, which reduces the migration and invasion of pancreatic cancer cells. Quercetin decreases TGF-β expression, inhibiting the phosphorylation and nuclear translocation of Smad2 and Smad3, and reducing the expression of Snail1, Zeb2, and c-Myc 112. In esophageal squamous cell carcinoma (ESCC), Chaperone-containing TCP1 subunit 6A (CCT6A) stimulates the TGF-β/Smad/c-Myc pathway, thereby promoting ESCC EMT and cell invasiveness 113.

### Transcription factors in TGF-β-mediated EMT

In addition to the important role of Smads signaling for EMT, many transcription factors have been widely reported to be associated with EMT. This regulation mainly involves three transcription factor families: Snail, Zeb, and bHLH family 93, 114. They regulate each other's expression, cooperate functionally on target genes, and play a central role in development, fibrosis, and cancer 115. Among them, Snail1/2, Zeb1/2, and Twist1/2 are considered to be implicated in the EMT process as master drivers 116.

#### Snail transcription factors

The Snail family are zinc finger proteins, including SNAIL (Snail1), SLUG (Snail2) and SMUC (Snail3). Snail promotes the EMT process by directly suppressing E-cadherin transcription through binding to the promoter sequence of the E-cadherin gene, E-box (5'-CACCTG) 117. In epithelial cells, the mRNA levels of E-cadherin and Snail are significantly negatively correlated 117. When the TGF-β signaling pathway is over-activated, Smad2 and Smad3 bound and activate Snail1/Snail2 118, 119, which suppresses E-cadherin, and Snail1/Snail2 continues to bind to the Snail1/Snail2 gene respectively and further suppresses E-cadherin expression 120. Additionally, Snail1/Snail2 can suppress the expression of other epithelial markers associated with epithelial phenotypes, such as Claudin, Occludin, and Cytokeratin 121, 122, while simultaneously increasing the expression of mesenchymal markers associated with mesenchymal phenotypes, such as fibronectin and vimentin 123. Studies have demonstrated that Snail family members have high expression in various cancer types, such as lung, breast, and colorectal malignancies 124-126. Increased levels of Snail impair cancer cell adhesion and facilitate tumor invasion and metastasis.

#### Zeb transcription factors

Zeb1 and Zeb2 belong to the human Zeb family of zinc finger proteins, which inhibit E-cadherin transcription by binding to regulatory gene sequences on the E-box 127. In addition, Zeb1 could interact with DNA methyltransferase 1 (DNMT1) through the Smad-binding domain and promote high methylation of the E-cadherin promoter region, which indirectly inhibits E-cadherin expression at the epigenetic level 128. Zeb1 and Zeb2 play a role in the TGF-β signaling pathway by interacting with Smad. However, they have contrasting regulatory functions in osteoblast development, implying that maintaining a balance between Zeb1 and Zeb2 may help regulate the TGF-β signaling pathway 129. Nevertheless, TGF-β triggers EMT in the majority of cancer cells and epithelial cells by activating Zeb1 and Zeb2 130. As an illustration, Zeb1 enhances the invasion of colorectal cancer by suppressing basement membrane (BM) gene expression 131.

#### Twist transcription factors

The bHLH family is a large family of transcription factors that control a wide range of developmental and pathological processes. Twist1 and Twist2 are highly conserved members of the Twist subfamily of bHLH transcription factors 132. Twist1 inhibits E-cadherin and promotes N-cadherin levels through E-box cis-acting elements, respectively 133, and also promotes EMT through up-regulation of vimentin 134. Twist1 directly activates Galectin-3 transcription and induces macrophage polarization towards the M2 phenotype, leading to the promotion of renal fibrosis 135. In prostate cancer, Twist1 regulates the transcriptional activity of CLU by binding to the distal promoter region of induces clusterin (CLU), which promotes TGF-β-mediated EMT and distant metastasis of the tumor 136. In breast cancer, TGF-β-induced EMT in HER2 cells is coordinated with the activation of the Wnt/β-catenin pathway. Under the action of TGF-β, Twist can directly promote the EMT process and also bind to the promoter of Wnt3 to activate the Wnt/β-catenin pathway and participate in Wnt/β-catenin pathway-mediated EMT 137.

### Non-coding RNAs in TGF-β-mediated EMT

In addition to the above-mentioned transcription factors that can regulate TGF-β-mediated EMT, non-coding RNAs (ncRNAs) are also involved in regulating the progression of EMT. Although ncRNAs do not directly encode proteins, they can regulate gene transcription and translation by interacting with specific messenger RNAs (mRNAs) 138, 139. Recent research has demonstrated that ncRNAs have a crucial function in tumor migration, invasion, and metastasis 140, 141. Specifically, some microRNAs, lncRNAs, and circRNAs have been identified as effectors of TGF-β-mediated EMT (Table 2) 142.

#### MicroRNAs

MicroRNAs (miRNAs) are ~22 nt small noncoding RNAs that can bind to 3′ UTR of their target mRNAs to suppress expression 143. It has been shown that miRNAs control the expression of EMT transcription factors, which affects TGF-β-mediated EMT. For example, miR-22, miR-30a, and miR-199a can directly target and inhibit Snail expression 144-146, while miR-141 targets NRP-1 and indirectly inhibits Snail expression 147, both of which inhibit the TGF-β-mediated EMT phenotype. In addition, miR-34a/b/c and miR-203 inhibited Snail expression, while Snail could bind miR-34a/b/c and miR-203 promoters to suppress its transcription, thus forming a dual negative feedback mechanism. However, frequent inactivation of miR-34a/b/c and miR-203 might disrupt the balance of these reciprocal regulations and promote TGF-β-mediated EMT 148, 149. Research shows that miRNA-200 significantly inhibits Zeb1 expression, and the enforced expression of the miR-200 alone was sufficient to prevent TGF-β-induced EMT. In contrast, ectopic expression of these microRNAs in mesenchymal cells initiates mesenchymal-to-epithelial transition (MET). Furthermore, in invasive breast cancer, lack of miR-200 expression is positively correlated with E-cadherin deficiency 150. In addition, miR-300 negatively regulates EMT by directly targeting Twist, inhibiting epithelial cancer cell invasion and metastasis 151. And miR-15a/16 inhibits prostate cancer cell invasion by targeting endogenous Smad3 and Acvr2a proteins to suppress Snail and Twist expression.

MiRNAs can also regulate TGF-β-mediated EMT by modulating the expression of epithelial or mesenchymal proteins 152. For instance, miR-145 and miR-497 promote E-cadherin and inhibit vimentin expression by inhibiting MTDH expression, suppressing the process of TGF-β-induced EMT in non-small cell lung cancer 153. In A549 lung cancer cells, overexpression of miR-23a inhibited E-cadherin expression and stimulated TGF-β-induced EMT 154. In addition, miR-199b-5p bound to the 3'-UTR of N-cadherin mRNA, which reduced N-cadherin expression in HCC cells and attenuated TGF-β1-induced EMT progression, thereby inhibiting metastasis and invasion of hepatocellular carcinoma cells 155. Another study showed that miR-194 could directly interact with the 3'-UTR of N-cadherin mRNA and reduce its expression in advanced gastric cancer cells 156.

#### LncRNAs

Long non-coding RNAs (lncRNAs) is a lengthy sequence of more than 200 nucleotides that controls the expression of specific genes by interacting with RNA, DNA, and proteins to form complexes. It is essential in various biological phenomena, including cell growth, differentiation, and metastasis 157, 158. Furthermore, lncRNAs are abnormally expressed in nearly all types of cancer and are linked to oncogenesis, metastasis, and tumor staging 159. And many reports have confirmed that lncRNAs are closely associated with TGF-β-mediated tumor EMT.

On the one hand, overexpression of lncRNAs in a variety of tumors induces EMT and promotes tumor metastasis. For example, the lncRNA LETS1 improves TGF-β-Smad signaling by stabilizing cell-surface TGF-β type I receptor (TβRI) to promote the migration of breast and lung cancer cells 160. LncRNA Smyca functions as a scaffold and facilitates the binding of Smad3 and Smad4, which promotes TGF-β/Smad signaling-mediated EMT and lung cancer cell migration 161. In a mouse colorectal cancer model, lncRNA LINC00941 and lncRNA Tug1 promote the migratory and invasive ability of colorectal cancer, thereby expediting lung metastasis. Mechanistically, LINC00941 directly binds to Smad4 protein and inhibits its degradation, thereby activating EMT 162, while the knockdown of Tug1 reduces Twist1 expression in CRC cells 163.

On the other hand, overexpression of certain lncRNAs in tumors inhibits TGF-β-induced EMT and tumor metastasis. For example, lncRNA LITATS1 inhibits EMT in breast cancer and non-small cell lung cancer cells by promoting polyubiquitination and proteasomal degradation of TβRI 164; lncRNA LINP1 inhibits TGF-β1/Smad4-induced EMT and cell invasion in lung cancer cells 165. In addition, lncRNA ANCR inhibited TGF-β1-induced EMT and breast cancer cell migration and metastasis by suppressing Runx2 166.

#### CircRNAs

Circular RNAs (circRNAs) are noncoding RNAs with tissue-specific and cell-specific expression patterns that form a covalently closed loop between the 5′ and 3′ ends 167. Aberrant expression of many circRNAs is observed in a wide range of cancers, suggesting that they play a crucial role in tumorigenesis and progression 168. This role may involve many different molecular mechanisms. The most common function of circRNAs is the ability to act as miRNA sponges, binding directly to the corresponding miRNAs and thereby regulating the expression of target genes. In addition, circRNAs are involved in transcriptional regulation, splicing, or even translating proteins 169. In recent years, many reports have shown that circRNAs are closely associated with TGF-β-mediated tumor EMT. For example, circITGB6 enhances IGF2BP3-mediated PDPN mRNA stability, which promotes TGF-β-induced EMT process and tumor metastasis 170. In addition, circPTK2 overexpression augmented TIF1γ expression and inhibited TGF-β-induced EMT and NSCLC cell invasion 171. CircRNAs not only function by regulating gene transcription and translation levels, but also modulate miRNA activity. In melanoma, circVANGL1 directly binds to miR-150-5p and promotes melanoma cell proliferation and invasion 172. In esophageal squamous cell carcinoma, circ-DOCK5 increased the stability of miR-627-3p, resulting in downregulation of Zeb1 and suppression of TGF-β-induced EMT 173.

## Conclusion

This review provides an overview of the crosstalk between cell adhesion mechanisms and TGF-β signaling pathway. Cell adhesion mechanisms not only facilitate the formation of tissue structures by promoting cell-cell or cell-ECM adhesions, but also participate in numerous non-adhesion signaling pathways. For example, CAMs can activate the TGF-β signaling pathway by binding to latent TGF-β and releasing mature TGF-β factors from ECM. This activation results in the transcription of downstream target genes, including Smad, c-Myc, Snail, Zeb, Twist, and non-coding RNAs. As a result, it in turn regulates the expression of CAMs or ECM remodeling, thus changing the cell adhesion ability. Currently, numerous studies have demonstrated a strong correlation between the TGF-β signaling pathway and the cell adhesion signaling pathway in cancer proliferation, migration, invasion, and immune evasion. Consequently, a range of drugs have been developed to specifically target crucial proteins involved in the TGF-β signaling pathway and cell adhesion signaling network. Various medications block the TGF-β signaling pathway by directly or indirectly inhibiting the production or function of TGF-β or TGF-β receptors. This includes small molecule inhibitors of TGF-β receptor kinase activity, TGF-β-directed antibodies, TGF-β ligand traps, antisense oligonucleotides (ASOs) targeting the TGF-β pathway, and vaccine-based approaches to modulate TGF-β signaling (these are well described in reviews 97, 174). Furthermore, there have been drugs specifically designed to target integrins 175. However, the development of drug research is not smooth sailing. The main shortcoming of antagonists targeting the TGF-β signaling pathway is the multifaceted function of TGF-β within the human body. In other words, TGF-β is necessary for healthy cells to control numerous essential physiological functions. Furthermore, there is a limited understanding of the dual opposite effects of TGF-β, which acts both as a tumor suppressor and a tumor promoter. Therefore, it is crucial to maintain rigorous control in clinical applications. Drugs that target integrins have a limited market due to the specificity of their target class, their pharmacokinetics, and the absence of clinical pharmacodynamic biomarkers. Hence, it is imperative to obtain further crosstalk between the TGF-β signaling pathway and the cell-adhesive signaling pathways. Hence, it is imperative to obtain further crosstalk between the TGF-β signaling pathway and the cell-adhesive signaling pathways. This will find novel regulatory factors that can be targeted for cancer treatment in the foreseeable future.

## Figures and Tables

**Table 1:** The association of CAMs and TGF-β signaling pathways

CAMs	Relationship with TGF-β signaling pathways	Ref.
**Integrins**		
αvβ6	Activate TGF-β and closely related to tumor invasion, metastasis, and poor prognosis in several human epithelial-derived malignancies	50, 51
αvβ8	Activate TGF-β and regulate the development and maturation of vascular and microglial cells	64, 65
**Cadherins**		
E-cadherin	TGF-β downregulates E-cadherin expression and disrupts cell-cell adhesion and epithelial integrity, which promotes the process of EMT	176
N-cadherin	TGF-β upregulates N-cadherin expression, which promotes the process of EMT	176
Cadherin-11	Promote TGF-β expression to induce the differentiation of MSCs into SMCs and regulate the ECM	71
FAT1	Promote TGF-β expression and form an immunosuppressive microenvironment, which facilitates the immune evasion of cancer	72
**IgSF**		
ICAMs	TGF-β promotes ICAMs expression and leads to neutrophil-mediated damage in lung injury	74
ALCAM	TGF-β induces the expression and shedding of ALCAM and promotes bone metastasis of prostate cancer	75
VCAM-1	TGF-β inhibits VCAM-1 expression in colorectal cancer vasculature, which allows tumor cells to avoid immunosurveillance	76
Jam-A	TGF-β induces the lysosomal pathway degradation of Jam-A, which allows breast cancer cells to acquire invasive cell properties	77, 78

**Table 2:** List of ncRNAs and their function in TGF-β-mediated EMT

Non-coding RNA	Targets gene	Cancer type	Role in TGF-β-mediated EMT	Regulation pathway	Ref.
MiR-15a/16	Smad3, ACVR2A	Prostate cancer	Suppressor	MiR-15a/16 targets and inhibits Smad3 and ACVR2A expression	177
MiR-22	Snail	Lung cancer	Suppressor	MiR-22 targets and inhibits the expression of Snail	144
MiR-23a	E-cadherin	Lung cancer	Promoter	MiR-23a inhibited E-cadherin expression and stimulated TGF-β-induced EMT	154
MiR-30a	Snail	Peritoneal fibrosis, liver fibrosis	Suppressor	MiR-30a targets and inhibits the expression of Snail	145, 146
MiR-34 a/b/c	Snail	Colorectal cancer	Suppressor	Snail and miR-34 form a dual negative feedback loop, inhibiting each other	148
MiR-141	Snail	Pancreatic cancer	Suppressor	MiR-141 targets NRP-1 to inhibit Snail expression	147
MiR-145 and miR-497	MTDH	Non-small cell lung cancer	Suppressor	MiR-145 and miR-497 attenuated MTDH expression by directly binding 3′-UTR of MTDH mRNA and exerting the tumor-suppression role	153
MiR-194	N-cadherin	Gastric cancer	Suppressor	MiR-194 inhibits N-cadherin expression	156
MiR-199a	Snail1, E-cadherin	Lung cancer	Suppressor	MiR-199a targeted and inhibited the expression of Snail and E-cadherin	178
MiR-199b-5p	N-cadherin	Hepatocellular carcinoma	Suppressor	MiR-23a inhibited the migration and invasion of HCC cells and suppressed tumor metastasis of xenografts in nude mice by targeting binding to N-cadherin	155
MiR-200	Zeb1, Zeb2	Breast cancer	Suppressor	MiR-200 targeted and inhibited the expression of Zeb	150
MiR-203	Snail2	Breast cancer	Suppressor	SNAI2 and miR-203 form a dual negative feedback loop, inhibiting each other's expression and thus controlling EMT	149
MiR-300	Twist	Breast cancer	Suppressor	MiR-300 directly targets Twist	151
LncRNA LETS1	TβRI	Breast cancer, lung cancer	Promoter	LncRNA LETS1 enhances TGF-β-Smad signaling by stabilizing TβRI on the cell surface	160
LncRNA Smyca	Smad3, Smad4	Breast cancer	Promoter	LncRNA Smyca acts as a scaffold, providing an additional binding surface for enhanced binding of Smad3 to Smad4	161
LncRNA LINC00941	Smad4	Colorectal cancer	Promoter	LncRNA LINC00941 inhibits Smad4 protein degradation by directly binding to Smad4 protein and inhibiting Smad4 protein degradation	162
LncRNA TUG1	Twist1	Colorectal cancer	Promoter	LncRNA TUG1 promotes Twist1 expression	163
LncRNA LITATS1	TβRI, SMURF2	Breast cancer, non-small cell lung cancer	Suppressor	LncRNA LITATS1 enhances polyubiquitination and proteasomal degradation of TGF-β type I receptor (TβRI)	164
LncRNA LINP1	—	Lung Cancer	Suppressor	Smad4 binds and inhibits lncRNA LINP1 expression and induces EMT and cell invasion in lung cancer cells	165
LncRNA ANCR	RUNX2	Breast cancer	Suppressor	LncRNA ANCR inhibits TGF-β1-induced EMT and breast cancer cell migration and metastasis of breast cancer cells by decreasing the expression of RUNX2	166
CircITGB6	PDPN	Liver cancer	Promoter	CircITGB6 promotes TGF-β-induced EMT and tumor metastasis through enhancing IGF2BP3-mediated PDPN mRNA stability	170
CircPTK2	TIF1γ	Non-small cell lung cancer	Suppressor	CircPTK2 overexpression augmented TIF1γ expression, inhibited TGF-β-induced EMT and NSCLC cell invasion	171
CircVANGL1	MiR-150-5p	Melanoma	Promoter	CircVANGL1 directly binds to miR‐150‐5p and promotes EMT of TGF‐β‐treated melanoma cells	172
Circ-DOCK5	MiR-627-3p	Esophageal squamous cell carcinoma	Suppressor	Circ-DOCK5 increased the stability of miR-627-3p resulting in downregulation of ZEB1 and suppression of TGF-β-induced EMT	173
